# Salinity gradients shape rhizosphere bacterial diversity and assembly of wheat in saline-alkali rice–wheat rotation soils

**DOI:** 10.3389/fmicb.2026.1931712

**Published:** 2026-08-28

**Authors:** Juntong Li, Ying Zhang, Quanquan Liu, Fengyu Jiang, Xufeng Liu, Jikun Xu, Ping Liu, Yan Wang, Chunhong Wu, Shuaipeng Zhao

**Affiliations:** 1 College of Biological and Pharmaceutical Engineering, Shandong University of Aeronautics, Binzhou, Shandong, China; 2 Dongying Natural Resources and Planning Bureau, Dongying, Shandong, China; 3 Haiyang Agricultural Technology Extension Center, Yantai, Shandong, China; 4 Shandong Province Engineering and Technology Research Center for Wild Plant Resources Development and Application of Yellow River Delta, Shandong University of Aeronautics, Binzhou, Shandong, China; 5 Shandong Key Laboratory of Eco-Environmental Science for the Yellow River Delta, Shandong University of Aeronautics, Binzhou, Shandong, China

**Keywords:** rhizosphere microorganism, rice-wheat cropping, saline-alkali land, salt regulation, wheat, Yellow River Delta

## Abstract

The saline-alkali soils of the Yellow River Delta constitute an important reserve of arable land resources in China, and the rice–wheat cropping system has been widely recognized as an effective approach for improving soil quality and increasing crop yields in this region. However, region-specific field investigations evaluating the effects of salinity on wheat rhizosphere bacterial communities within this system remain limited. To fill this research gap, we conducted a field salinity gradient experiment with low-salinity (LST, 1.5 g/kg), medium-salinity (MST, 3.0 g/kg), and high-salinity (HST, 5.0 g/kg) treatments to explore how salt levels alter soil physicochemical traits and rhizosphere bacterial assemblages. We found rising salinity degraded soil fertility: compared with LST, MST and HST decreased soil pH by 0.73 and 0.70 units, organic matter by 35.4% and 68.5%, ammonium nitrogen by 54.2% and 46.5%, and sucrase activity by 82.6% and 72.7%. Available phosphorus reached the lowest level under MST, decreasing by 41.8% and 40.3% vs. LST and HST, while nitrate nitrogen peaked at HST with increments of 20.8% and 22.0% relative to LST and MST. OTU analysis showed continuous transitional succession of microbiota along the salinity gradient. Elevated salinity boosted the relative abundance of dominant phyla including Proteobacteria and Bacteroidota, as well as genera *Thiobacillus* and *Confluentibacter*, while suppressing the abundance of Actinobacteriota and its representative genus *Nocardioides*. At the genus level, unclassified bacterial taxa dominated all treatments and increased in relative abundance with rising salinity, representing valuable untapped microbial resources in saline–alkali wheat rhizosphere soils. Redundancy analysis verified ammonium nitrogen strongly correlated with community composition, and LEfSe distinguished *Flavobacterium, Sulfurimonas*, and *Pseudomonas* as characteristic high-salinity-enriched taxa.

## 1 Introduction

Food security is a cornerstone of national strategy, and saline–alkali land represents an important reserve of arable land resources (Li, 2025). According to incomplete statistics from UNESCO and FAO, approximately 950 million hectares of saline–alkali land exists worldwide, of which about one-tenth is in China (Zhang et al., 2006). At present, China possesses 3.6 × 10^7^ hm^2^ of saline-alkali land across different categories (Yang, 2008). As a typical coastal saline–alkali region, the Yellow River Delta contains approximately 465.7 thousand hectares of saline–alkali land (Yang et al., 2023). The total land area of the Yellow River Delta is 2.15 million hectares, with cultivated land totaling 786,000 hectares, with approximately 700,000 hectares of uncultivated land resources remaining. It is rich in natural resources, possessing a unique ecological environment, and benefiting from favorable climatic conditions, it stands as a rare, undeveloped reserve resource treasure trove along China's eastern coast (Dong, 2010). Currently, crop production in this area is dominated by single-cropping rice, single-cropping cotton, and wheat–maize double cropping (Liu et al., 2023). These systems are associated with severe soil salt accumulation, low land-use efficiency, and relatively low crop yields (He et al., 2020; Yin et al., 2024).

To address the current challenges of crop cultivation in saline–alkali soils of the Yellow River Delta, the Biological Breeding and Aerospace Biotechnology team of Shandong Aviation University introduced a rice–wheat double-cropping system. In 2025, the total grain yield of the rice–wheat system reached 15,563.4 kg·hm^−2^, achieving a “ton-grain field” standard under moderately saline–alkali conditions in the Yellow River Delta. This practice effectively improved soil quality, increased grain yield, and enhanced the productivity of saline–alkali lands. Research shows that after rice harvest, drainage and field drying trigger a sharp transition from reducing to oxidizing conditions (de Freitas et al., 2024), which not only alters nutrient availability but also imposes strong selective pressure on soil microbial communities. In addition, flooding irrigation exerts dual effects on salt regulation: on the one hand, it leaches soluble salts from the plow layer and creates a more favorable growth environment for subsequent crops; on the other hand, water evaporation may drive upward migration of deep-layer salts and their accumulation in the surface soil, especially in poorly drained areas (Xu et al., 2020). In the rice-wheat rotation system, irrigation and fertilization practices during the rice season exert short-term yet significant legacy effects on the soil bacterial communities of the subsequent wheat season (Tian et al., 2022). In this system, rice is well adapted to flooded saline conditions with mature cultivation practices, while wheat's inherently low saline-alkali tolerance makes it the primary limiting factor for annual productivity (Ma and Geng, 2026; Zhang et al., 2026). However, no study has investigated the response patterns of wheat rhizosphere microbiota to salinity gradients in the context of rice-season legacy effects in rice–wheat rotation systems.

Soil salinization constitutes one of the primary constraints on agricultural development in the saline-alkali lands of the Yellow River Delta. Salinity acts as a key limiting factor, directly inhibiting crop growth and development, reducing yields, and consequently constraining regional agricultural productivity (Song et al., 2017). Diverse microbial communities colonize both the aboveground and belowground organs of crops. Numerous microorganisms enhance plant physiological and metabolic functions and improve environmental adaptability through interactions with their host plant (Chen et al., 2022). As the core microdomain of plant-soil interactions, the rhizosphere selectively recruits soil microorganisms according to plant growth and developmental demands. The complex and dynamic interactions between plants and soil microorganisms under abiotic stress can promote plant growth, alleviate stress-induced damage, and minimize environmental impacts (Fouad et al., 2025). In saline-alkali environments, the rhizosphere microbial community functions as the primary biological barrier against stress, playing an irreplaceable role in maintaining plant productivity (Fouad et al., 2025).

Previous studies have shown that wheat rhizosphere microbial communities not only adapt to high salinity and alkalinity but also assist plants in mitigating salt-induced damage by enhancing stress resistance and adaptive response (Sharma et al., 2024). As the most abundant and diverse group within the rhizosphere microbiome, bacteria play a pivotal role in promoting nutrient uptake and improving stress tolerance in crops. Soil salinity has been identified as a major environmental stressor influencing wheat rhizosphere bacterial communities (Fouad et al., 2025). Conclusions regarding the effects of salinity regulation on rhizosphere bacterial community diversity vary. Some researchers suggest that high salinity significantly reduces rhizosphere bacterial diversity. Junpeng Liu's et al. s study on wild soybean *(Glycine soja)* showed that salt stress significantly reduced the diversity of rhizosphere bacterial communities and changed the community structure (Liu et al., 2025). In contrast, Hui Yang et al. observed that the diversity of bacterial communities in the rhizosphere soil of Jerusalem artichoke *(Helianthus tuberosus)* exhibits an increasing trend within a certain range of increasing salinity (Yang et al., 2016). Furthermore, existing research predominantly focuses on overall soil bacterial communities, with insufficient investigation into the unique characteristics of the “wheat rhizosphere” microenvironment (Yahya et al., 2021).

As an efficient agricultural model, the rice–wheat cropping system fully utilizes the light and heat resources of the Yellow River Delta, increases cropping intensity and land-use efficiency, and achieves the combined ecological and economic benefits of soil improvement and yield enhancement. Nevertheless, research on the specificity of this system in the coastal saline–alkali soils of the Yellow River Delta remains limited, particularly with respect to the adaptive mechanisms of the wheat rhizosphere microenvironment under different salinity regulation gradients. Therefore, this study focused on the coastal saline-alkali rice-wheat cropping system in the Yellow River Delta and investigated the effects of salinity regulation on wheat rhizosphere bacterial community composition, diversity, and potential functional characteristics through comprehensive analysis of bacterial community structure and diversity combined with key soil physicochemical properties (pH, organic matter, available nutrients, etc.). The aim was to clarify the response patterns of wheat rhizosphere bacterial communities under different salinity conditions. This study provides a theoretical foundation for the targeted development of healthy soils through microbial approaches and for the optimization of high-yield rice–wheat cropping systems and supporting management practices in coastal saline–alkali farmland of the Yellow River Delta.

## 2 Materials and methods

### 2.1 Experimental field overview

The study area is situated at the Dongying Molecular Design Breeding Research Center of the Chinese Academy of Sciences, Huanghekou Town, Kenli District, Dongying City, Yellow River Delta (118°50′24″E, 37°41′24″N). This region has a temperate monsoon climate with hot and rainy summers and cold and dry winters. The average wind speed is approximately 3–4 m/s, with prevailing northeasterly and northwesterly winds. The average annual precipitation is about 550–650 mm, mainly concentrated in July–September. The average annual evaporation is around 1,500–2,000 mm, and the frost-free period is approximately 180–200 days. Before the initiation of the experiment (pre-cultivation), the soil in the experimental area was moderately alkaline, with an average pH of 8.58, classified as sulfate-chloride saline-alkali soil. The soil properties were as follows: organic matter 14.70 g/kg; total nitrogen 1.12 g/kg; available potassium 130.81 mg/kg; available phosphorus 28.51 mg/kg; and soil texture was sandy clay loam. After rice harvest (prior to wheat sowing), the soil in the experimental area was moderately alkaline, with an average pH of 8.65, classified as sulfate-chloride saline-alkali soil. The soil properties were as follows: organic matter 11.18 g/kg; total nitrogen 0.71 g/kg; available potassium 16.63 mg/kg; available phosphorus 35.93 mg/kg; and soil texture was sandy clay loam.

### 2.2 Experimental design

This experiment was conducted under a rice-wheat rotation system in the field after rice harvest. After rice harvest, the experimental field was divided into nine plots. One-way analysis of variance (ANOVA) was performed on pre-experimental soil samples to verify no significant difference (*p* > 0.05) in initial soil properties among replicate plots. Three soil salinity gradients were established via artificial addition of sulfate-chloride mixed salt matching the salt composition of native soil: low salinity, medium salinity, and high salinity. The actual total salt contents in the 0-20 cm topsoil were 1.5 g/kg, 3.0 g/kg, and 5.0 g/kg, respectively, corresponding to salinity ranges of 1–2‰, 2–4‰, and 4-6‰. Each treatment included three biological replicates: low-salinity treatment (LST1, LST2, LST3), medium-salinity treatment (MST1, MST2, MST3), and high-salinity treatment (HST1, HST2, HST3), resulting in a total of 9 experimental plots. Each plot covered an area of 10 m^2^ (2 m × 5 m). Ridges (30 cm in height and 40 cm in width) were built between adjacent plots, and a 50 cm wide aisle was reserved between plots for field management operations. Two rows of crops were planted around each plot as guard rows to eliminate edge effects. Rice cultivar Yanfeng 47 and wheat cultivar Jimai 22 were sown in their respective growing seasons. Wheat was sown approximately 10 days after rice harvest. No tillage, leaching, drainage or other field management practices were applied during this interval. All experimental plots received uniform conventional field management to eliminate interference from agronomic practices. Soil salinity dynamics were monitored regularly throughout the experiment, and water control was adopted to maintain the stability of the salinity gradient.

### 2.3 Measurement items and methods

#### 2.3.1 Sample collection

At wheat maturity, five sampling points were selected from each experimental plot using the diagonal sampling method. Wheat plants were carefully excavated with a shovel to avoid root damage and preserve root integrity. Sterilized paper was spread on the ground, and soil within 1 cm of the root surface was gently brushed off using a sterile brush to collect rhizosphere soil. For each plot, the five subsamples were thoroughly mixed to form one composite rhizosphere soil sample representing that plot. Each composite sample was divided into two portions: one portion was air-dried in the laboratory, ground, and sieved through a 20-mesh sieve for determination of basic soil physicochemical properties; the other portion was stored in an ice box at −20 °C during transport and immediately transferred to a −80 °C freezer upon return to the laboratory for subsequent high-throughput sequencing analysis (Zhang Y. et al., 2024).

#### 2.3.2 Determination of basic soil physicochemical properties

Soil pH was determined using a pH meter with a water-to-soil ratio of 5:1. Organic matter content was measured by the potassium dichromate-concentrated sulfuric acid heating method. Ammonium nitrogen content was determined via indophenol blue spectrophotometry, nitrate nitrogen content by naphthyl ethylenediamine dihydrochloride colorimetry, available phosphorus content by quinoline phosphomolybdate colorimetry, and available potassium content by 1 mol/L ammonium acetate extraction-flame photometry (Bao, 2000). Sucrase activity was assayed using the 3,5-dinitrosalicylic acid colorimetric method at λ = 508 nm (Guan, 1986).

#### 2.3.3 Determination of cultivable microbial counts in soil

Viable colony-forming units (CFUs) of cultivable bacteria and fungi in the rhizosphere soil were determined using the dilution plate method. A soil suspension was prepared by shaking 10 g of fresh soil in 90 mL of sterile physiological saline for 30 min. The suspension was subjected to tenfold serial dilutions to 10^5^. Aliquots of the diluted suspensions were spread onto beef extract peptone agar (BEPA) for bacteria and potato dextrose agar (PDA) for fungi and incubated at 37 °C and 28 °C for 3–7 days, respectively. CFUs per kilogram of soil were calculated based on colony counts and the corresponding dilution factors.

#### 2.3.4 Rhizosphere bacteriome molecular analysis

Soil samples stored at −80 °C were transferred into 1.5-mL Eppendorf tubes for subsequent bacterial community sequencing, covering three biological replicates of each salinity treatment.The V3–V4 region of the 16S RNA gene was amplified by PCR using the primers: F, ACTCCTACGGGAGGCAGCA; R, GGACTACHVGGGTWTCTAAT (Shaffer et al., 2022). The 25 μL PCR reaction system contained 12.5 μL 2 × Taq PCR MasterMix, 1 μL of each forward (5 → w) and reverse primer (10 μM), 3 μL template DNA, and 7.5 μL nuclease-free water. The thermal cycling program was set as follows: initial denaturation at 95 °C for 5 min; 28 cycles of denaturation at 95 °C for 45 s, annealing at 55 °C for 50 s, extension at 72 °C for 45 s; followed by a final extension at 72 °C for 10 min. The amplified products were purified using an Omega DNA purification kit (Omega Inc., Norcross, GA, USA) and quantified with a Qsep-400 system (BiOptic Inc., New Taipei City, Taiwan, China). The amplicon library was paired end sequenced (2 × 250 bp) on an Illumina NovaSeq 6,000 platform (Beijing Biomarker Technologies Co., Ltd., Beijing, China).

### 2.4 Data analysis and processing

Statistical analyses were performed using SPSS (IBM SPSS, Chicago, IL, USA). Prior to parametric testing, the normality and homogeneity of variance for all datasets (including soil properties, culturable microbial counts, and alpha diversity indices) were verified using the Shapiro-Wilk and Levene's tests, respectively. One-way analysis of variance (ANOVA) followed by least significant difference (LSD) post-hoc test was applied to determine significant differences among treatments for soil physicochemical properties, colony-forming units (CFUs), and alpha diversity indices, with statistical significance set at *p* < 0.05.

Raw 16S rRNA sequencing data were first quality-trimmed using Trimmomatic v0.33 with a 50 bp sliding window and a minimum average Phred quality score of 20 under the Phred33/64 system (Bolger et al., 2014), and primers were removed with Cutadapt v1.9.1 at a maximum mismatch rate of 20% and a minimum coverage of 80% (Martin, 2011). Paired-end reads were assembled in USEARCH v10.0 with a minimum overlap of 10 bp, a minimum overlap similarity of 90%, and a default maximum mismatch of 5 bp (Edgar, 2013). Chimeric sequences were filtered using UCHIME v8.1, and sequences sharing >80% local similarity with two parent sequences were defined as chimeras and removed (Edgar et al., 2011). OTU clustering was performed at 97% sequence similarity using USEARCH v10.0, and low-abundance OTUs were filtered at a threshold of 0.005% of the total sequences to generate a standardized OTU table (Edgar, 2013; Bokulich et al., 2013).

Alpha diversity indices were calculated using QIIME2 (version 2020.6) (Bolyen et al., 2019). ANOVA was applied to compare differences in soil properties and Alpha diversity indices among groups. Principal coordinate analysis (PCoA) and non-metric multidimensional scaling (NMDS) were performed using R software (version 3.4.4) (Some, 1966). Beta diversity indices were also analyzed by ANOVA in SPSS. The Chao1 and ACE indices reflected species richness based on species abundance, while the Shannon and Simpson indices characterized species diversity, which was affected by both species richness and community evenness. Linear discriminant analysis effect size (LEfSe) analysis was used to identify key bacterial biomarkers responsible for differentiating community compositions among groups (Segata et al., 2011). Prior to interpreting PERMANOVA results, the homogeneity of multivariate dispersions was assessed using the PERMDISP test (betadisper function in the vegan package) based on Bray-Curtis dissimilarities with 999 permutations. Permutational multivariate analysis of variance (PERMANOVA) was conducted to test differences in microbial community structure, with significance defined as *p* < 0.05. Redundancy analysis (RDA) of soil bacterial communities in relation to environmental variables was performed in R software (version 3.4.4) using the vegan 2.3-0 package. For redundancy analysis (RDA), variance inflation factor (VIF) was calculated for all environmental variables to diagnose multicollinearity. Variables with VIF > 10 were stepwise excluded to avoid model overfitting. The adjusted *R*^2^ was reported to present the corrected explanatory power of the model. Functional annotation of bacterial communities was carried out using the FAPROTAX database (version 1.2.6) (Louca et al., 2016). PICRUSt2 was applied to predict KEGG functional profiles, which were then integrated with the microbial biomarkers identified by LEfSe (Zhang G. et al., 2024).

## 3 Results

### 3.1 Soil physiochemical properties under different salinity gradient

Table 1 shows the physicochemical properties of rhizosphere soils under different treatments. Among different salinity gradients, only available potassium content showed no significant difference (*P* > 0.05), while all other indicators exhibited significant effects (*P* < 0.05). Compared to low-salinity conditions, medium- and high-salinity treatments reduced soil pH by 0.73 and 0.70 units, respectively. Salinity significantly affected soil organic matter content (*P* < 0.05), showing a continuous decline with increasing salinity. Medium- and high-salinity treatments reduced organic matter content by 35.4% and 68.5%, respectively, compared to low salinity. Available phosphorus content was lowest under medium-salt treatment, decreasing by 41.8% and 40.3% compared to low- salinity and high- salinity treatments, respectively. Soil available potassium content showed no significant differences among treatments (*P* > 0.05), ranging from 146.50 to 150.55 mg/kg. Ammonium nitrogen content peaked under low-salinity conditions, decreasing by 54.2% and 46.5% under medium- and high-salinity treatments, respectively. Nitrate nitrogen content reached its highest value under high-salinity treatment, increasing by 20.8% and 22.0% compared to low- and medium-salinity treatments, respectively. Under controlled salinity levels, sucrase activity exhibited significant differences among treatment groups (*P* < 0.05). Compared with the low- salinity treatment, both medium- salinity and high- salinity treatments significantly decreased sucrase activity (*P* < 0.05), with significant reductions of 82.6% and 72.7% recorded for the two treatments, respectively.

**Table 1:** Changes of soil physicochemical properties among groups.

Groups	pH	OM (g/kg)	AP (mg/kg)	AK (mg/kg)	${\text{NH}}_{4}^{+}$ -N (mg/kg)	${\text{NO}}_{3}^{-}$-N (mg/kg)	Sucrase [mg/(g·d)]
LST	8.42 ± 0.15a	13.91 ± 0.89a	65.67 ± 2.45a	146.50 ± 0.46a	122.90 ± 1.38a	84.17 ± 4.21b	69.66 ± 0.62a
MST	7.69 ± 0.22b	8.98 ± 0.57b	38.25 ± 3.05b	149.20 ± 2.59a	56.35 ± 10.91b	83.36 ± 4.67b	12.12 ± 0.87c
HST	7.72 ± 0.02b	4.38 ± 1.67c	64.10 ± 1.21a	150.55 ± 4.64a	65.78 ± 3.64b	101.68 ± 4.22a	19.00 ± 2.20b

OM, AP, AK, ${\text{NH}}_{4}^{+}$ -N, ${\text{NO}}_{3}^{-}$ -N respectively represent organic matter, available phosphorus, available potassium, ammonium-nitrogen, and nitrate-nitrogen. Values are expressed as means ± standard error. Different lowercase letters within the same column indicate significant differences at the 0.05 level. LST: the rhizosphere soil samples of low-salinity treatment; MST: the rhizosphere soil samples of medium-salinity treatment; HST: the rhizosphere soil samples of high-salinity treatment.

### 3.2 Cultivable microbial populations in soil

**Figure 1:**
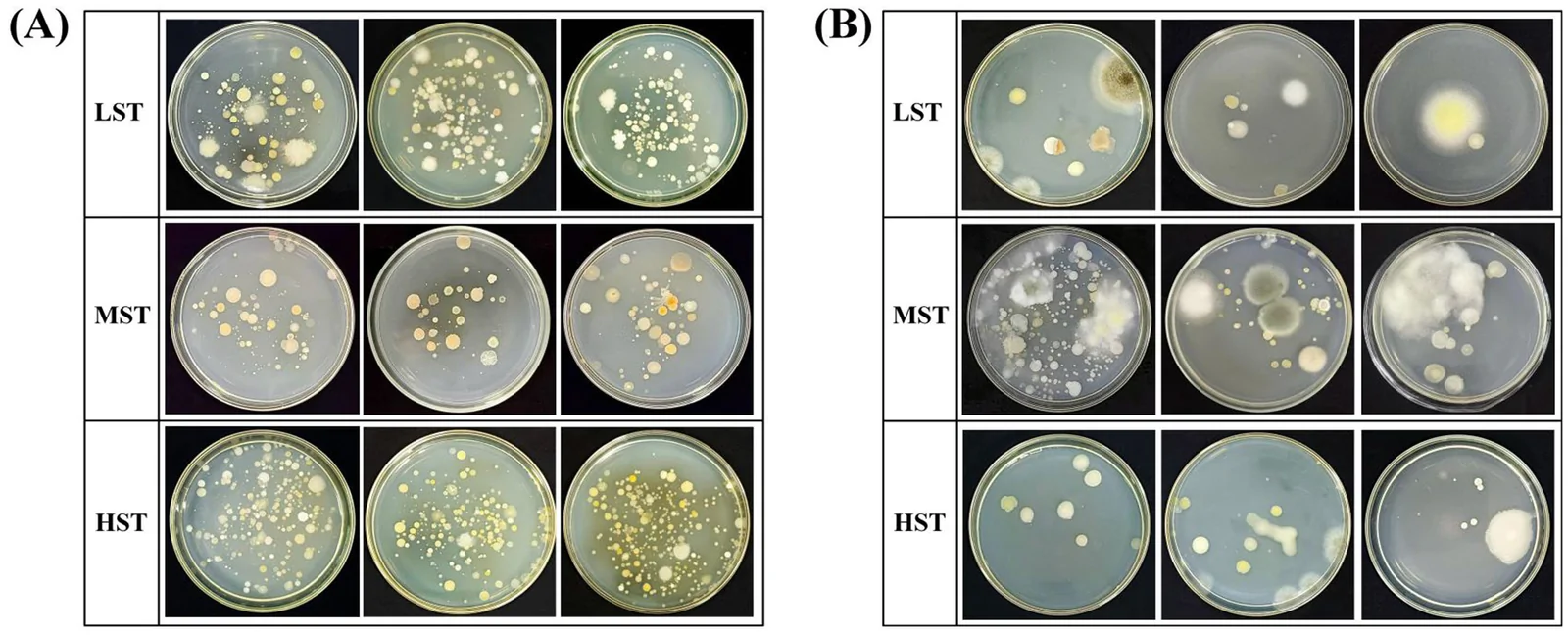
Cultivable CFU counts at a dilution factor of 10^3^ among different groups **(A):** bacterial CFU counts; **(B)** fungal CFU counts). Rows in the table represent three replicate groups established under each salinity gradient. LST: the rhizosphere soil samples of low-salinity treatment; MST: the rhizosphere soil samples of medium-salinity treatment; HST: the rhizosphere soil samples of high-salinity treatment.

Culturable microbial CFU counts as vital indicators for evaluating soil microbial abundance. Significant differences in both bacterial and fungal colony counts were observed across the salinity treatments (*P* < 0.05; Table 2). Compared with the low-salinity treatment, the medium-salinity treatment significantly decreased bacterial CFU counts by 58.3% (*P* < 0.05), while the high-salinity treatment significantly increased bacterial count by 17.3% (*P* < 0.05). Fungal CFU counts in the MST treatment were significantly higher than those in both LST and HST treatments *(P* < 0.05), while no significant difference was detected between LST and HST (*P* > 0.05). Figure 1 shows cultivable CFU counts obtained at a dilution factor of 10^3^.

**Table 2:** CFU counts of rhizosphere soil microorganisms.

Groups	Bacterial ( × 10^1^^1^ CFU/kg)	Fungal ( × 10^9^ CFU/kg)
LST	1.39 ± 0.05b	3.00 ± 0.10b
MST	0.58 ± 0.02c	5.00 ± 0.17a
HST	1.63 ± 0.05a	3.00 ± 0.09b

Values are expressed as means ± standard error (n = 3 independent biological replicates per treatment). Different lowercase letters within the same column indicate significant differences at the 0.05 level.

### 3.3 Analysis of differences in alpha diversity indices among groups

**Figure 2:**
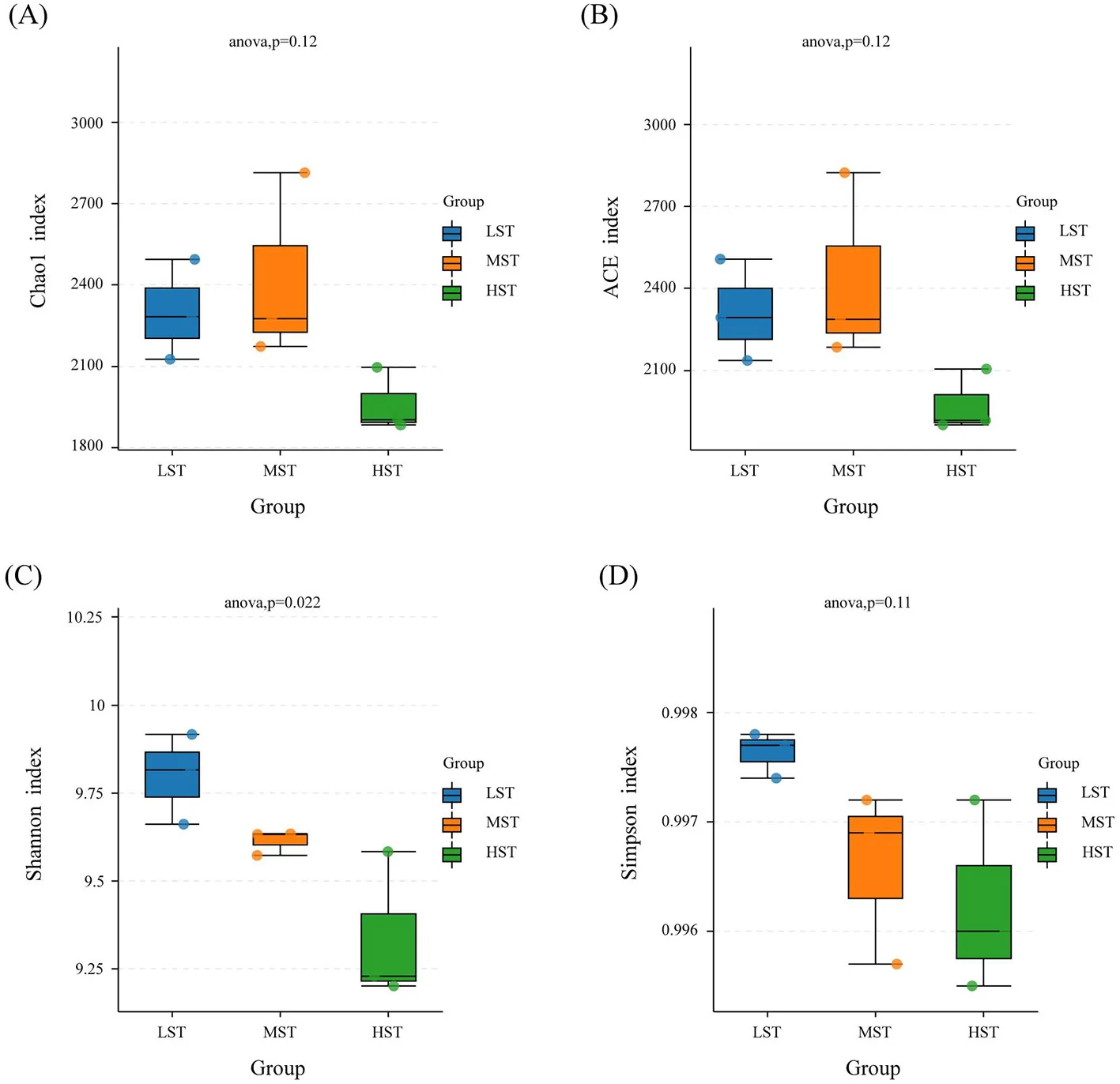
Alpha diversity indices of rhizosphere bacterial communities under three salinity treatments (LST: low salinity; MST: medium salinity; HST: high salinity). **(A)** Chao1 index, **(B)** ACE index, estimating species richness; **(C)** Shannon index and **(D)** Simpson index, reflecting community diversity. The *P* values of one-way ANOVA are displayed at the top of each panel. Dots represent biological replicates, boxes indicate the interquartile range, and horizontal lines inside boxes denote median values.

Figure 2 shows the differences in alpha diversity indices among treatments. Overall, salinity exerted stronger effects on community diversity and evenness than on richness, with only the Shannon index showing significant differences (*P* < 0.05), while the remaining indices did not differ significantly (*P* > 0.05). With increasing salinity, the Chao1 and ACE indices first increased and then declined, whereas the Simpson and Shannon indices showed a decreasing trend. These results indicate that community richness was highest under medium salinity and lowest under high salinity, while community diversity gradually declined with increasing salinity.

### 3.4 OTU abundance analysis

**Figure 3:**
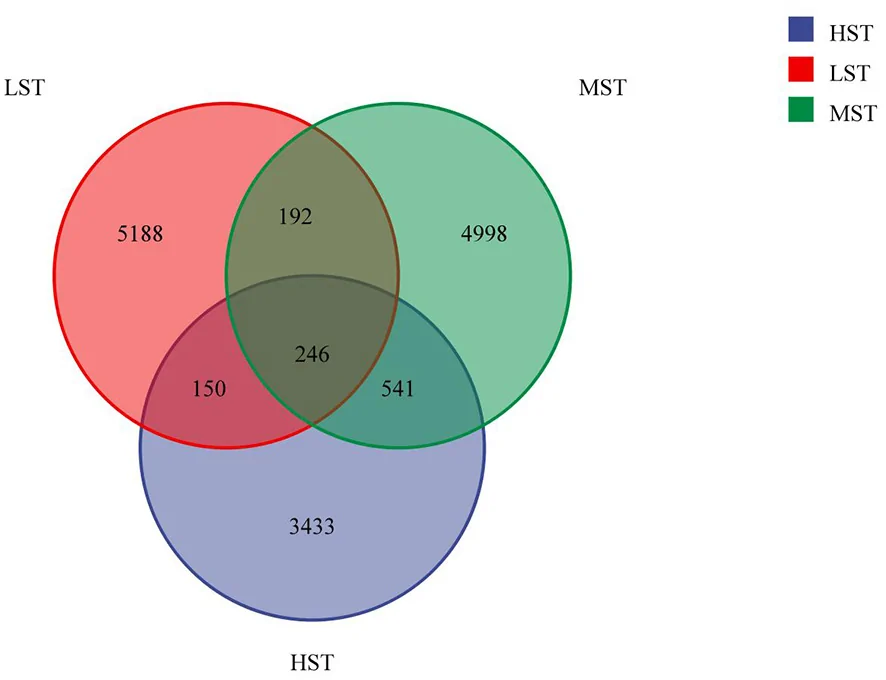
Venn diagram showing the numbers of shared and unique OTUs among different groups. Each circle represents a treatment group. Values in overlapping regions indicate shared OTUs, whereas values in non-overlapping regions indicate unique OTUs.

Based on operational taxonomic unit (OTU) abundance, Venn diagram analysis was performed to compare soil bacterial community composition under different salinity treatments (Figure 3). The total numbers of OTUs under low-, medium-, and high-salinity treatments were 5,776, 5,977, and 4,370, respectively, with 5,188, 4,998, and 3,433 unique OTUs in each treatment. A total of 246 OTUs were shared among all three treatments. These results suggest that although bacterial communities across salinity gradients exhibited high specificity, they also maintained a certain degree of similarity. The high-salinity treatment reduced both the total number of OTUs and the number of unique OTUs. Pairwise comparisons showed that the medium- and high-salinity treatments shared 541 OTUs, which was substantially higher than the numbers shared between medium- and low-salinity treatments (192 OTUs) and between low- and high-salinity treatments (150 OTUs). The higher number of shared OTUs between the MST and HST groups (541) compared to other pairwise combinations suggests a potential transitional role for the medium-salinity environment in community succession.

### 3.5 Analysis of soil microbial community structure

**Figure 4:**
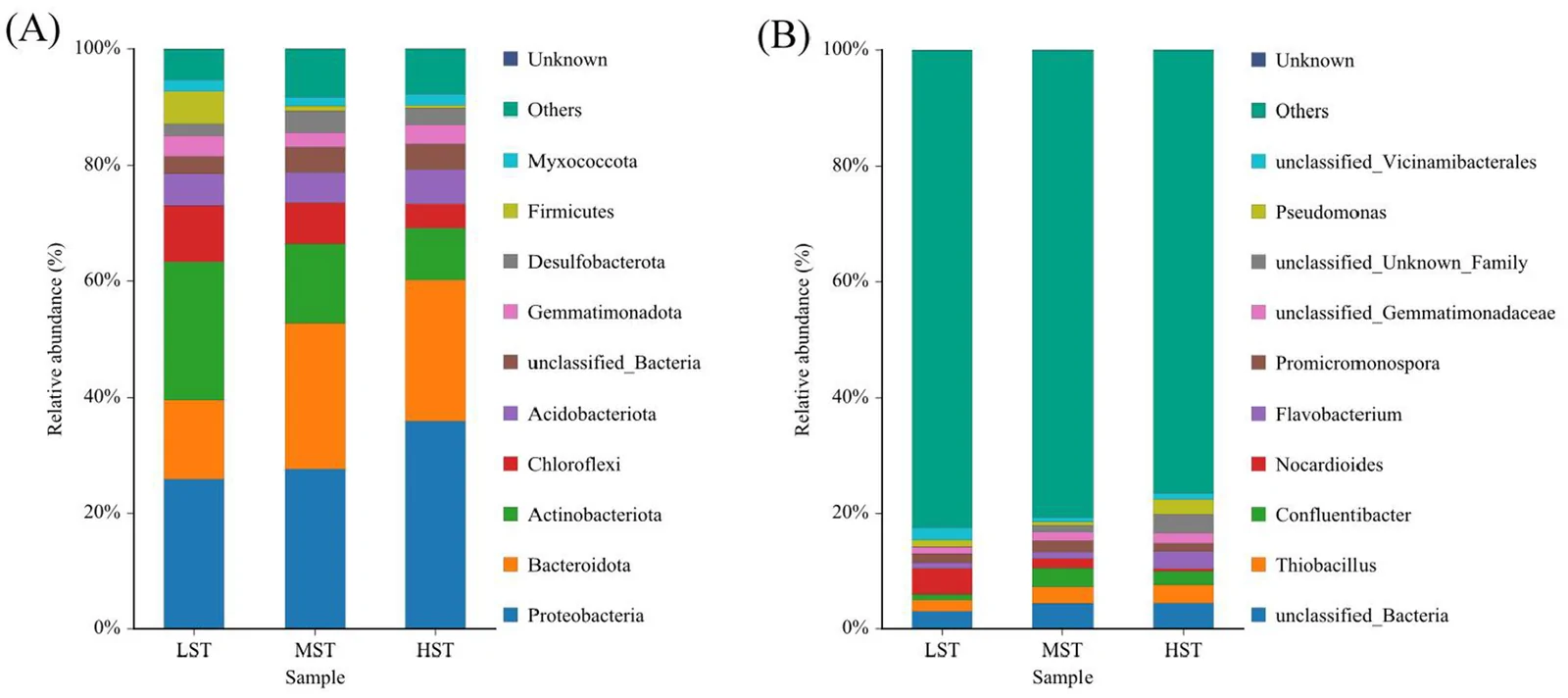
Relative abundance of the top 10 dominant bacterial taxa under low (LST), medium (MST) and high salinity (HST) treatments. **(A)** Taxonomic composition at the phylum level; **(B)** Taxonomic composition at the genus level. “Others” represents the collective relative abundance of taxa outside the top 10 classified groups. The vertical axis shows relative abundance expressed as a percentage.

Different salinity levels significantly influenced the relative abundance of dominant bacterial taxa (Figure 4). At the phylum level, Proteobacteria exhibited the highest relative abundance (25.9%−35.9%), followed by Bacteroidota and Actinobacteriota (Figure 4A). The relative abundance of Actinobacteriota decreased with increasing salinity, whereas Proteobacteria and Bacteroidota showed increasing trends. At the genus level (Figure 4B), unclassified bacteria had the highest relative abundance, which increased with rising salinity. Compared with the low-salinity treatment, the medium- and high-salinity treatments increased the relative abundance of *Thiobacillus* by 0.9% and 1.2%, respectively, and that of *Confluentibacter* by 2.3% and 1.4%, respectively. In contrast, the relative abundance of *Nocardioides* declined with increasing salinity, decreasing by 2.9% and 4.0% under medium- and high-salinity treatments, respectively, compared with low salinity. Full relative abundance data of bacterial taxa at the phylum and genus levels across all salinity treatments are provided in Supplementary file 1.

### 3.6 Differences in bacterial community composition across salinity treatments

**Figure 5:**
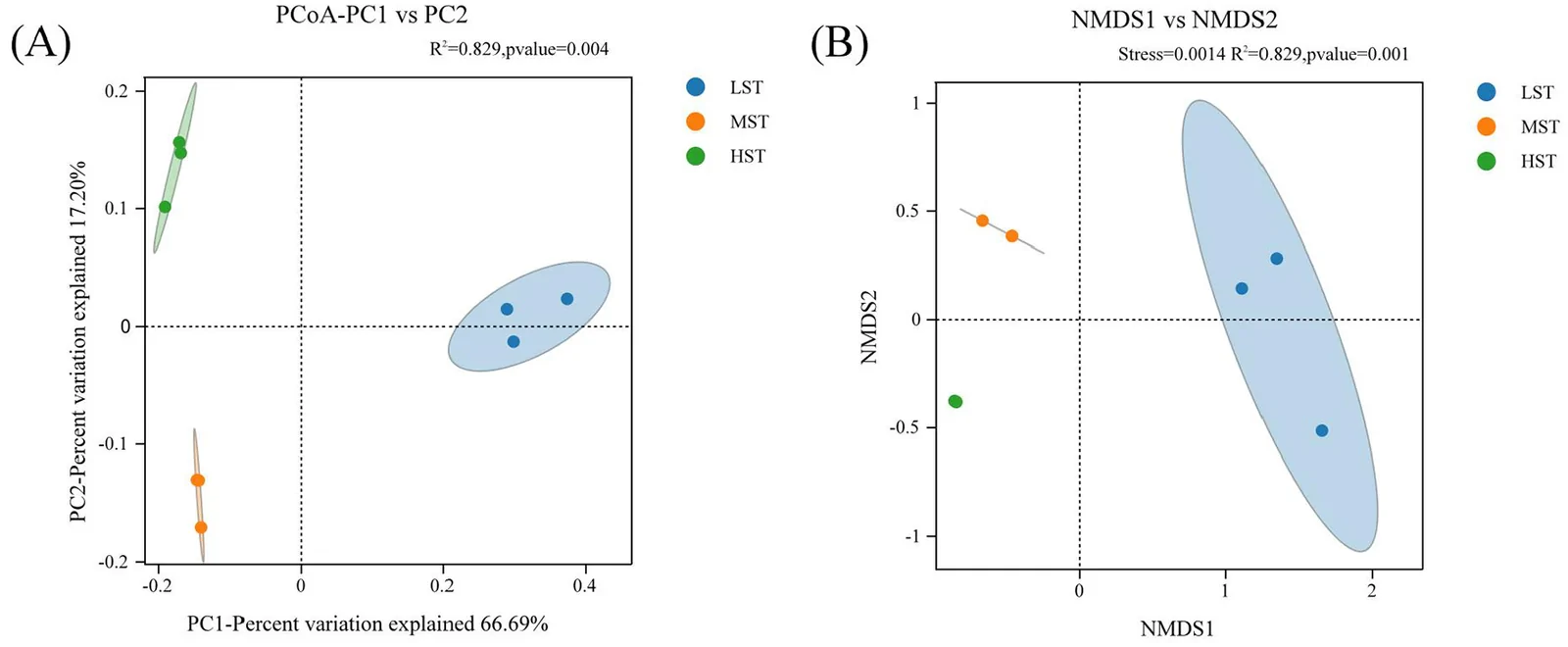
Beta diversity analysis based on Bray-Curtis distance showing changes in bacterial community structure **(A)** PCoA and PERMANOVA analysis; **(B):** NMDS and PERMANOVA analysis).

PCoA revealed distinct clustering patterns of bacterial community structures along the PC1 and PC2 axes. PC1 and PC2 explained 66.69% and 17.20% of the total variance, respectively, accounting for 83.89% cumulatively. No overlap was observed among groups along either axis, and samples within each group clustered closely, indicating clear intergroup differences and strong intragroup consistency (Figure 5A). These results were further supported by non-metric multidimensional scaling (NMDS) analysis (Stress = 0.0014). In the NMDS1–NMDS2 space, the three treatment groups were clearly separated, and the clustering patterns were consistent with those observed in the PCoA analysis (Figure 5B).

PERMANOVA results demonstrated highly significant differences among groups (PCoA: *P* = 0.004; NMDS: *P* = 0.001), with treatment explaining 82.9% of the total variation in community structure (R^2^ = 0.829). These findings confirm that salinity gradients were the primary drivers of changes in bacterial community structure. The PERMDISP test showed homogeneous multivariate dispersions among the three salinity treatments (*F* = 1.342, *p* = 0.325), confirming that the significant PERMANOVA result was driven by differences in community centroids rather than dispersion heterogeneity.

### 3.7 Correlation between soil bacterial diversity and environmental factors

**Figure 6:**
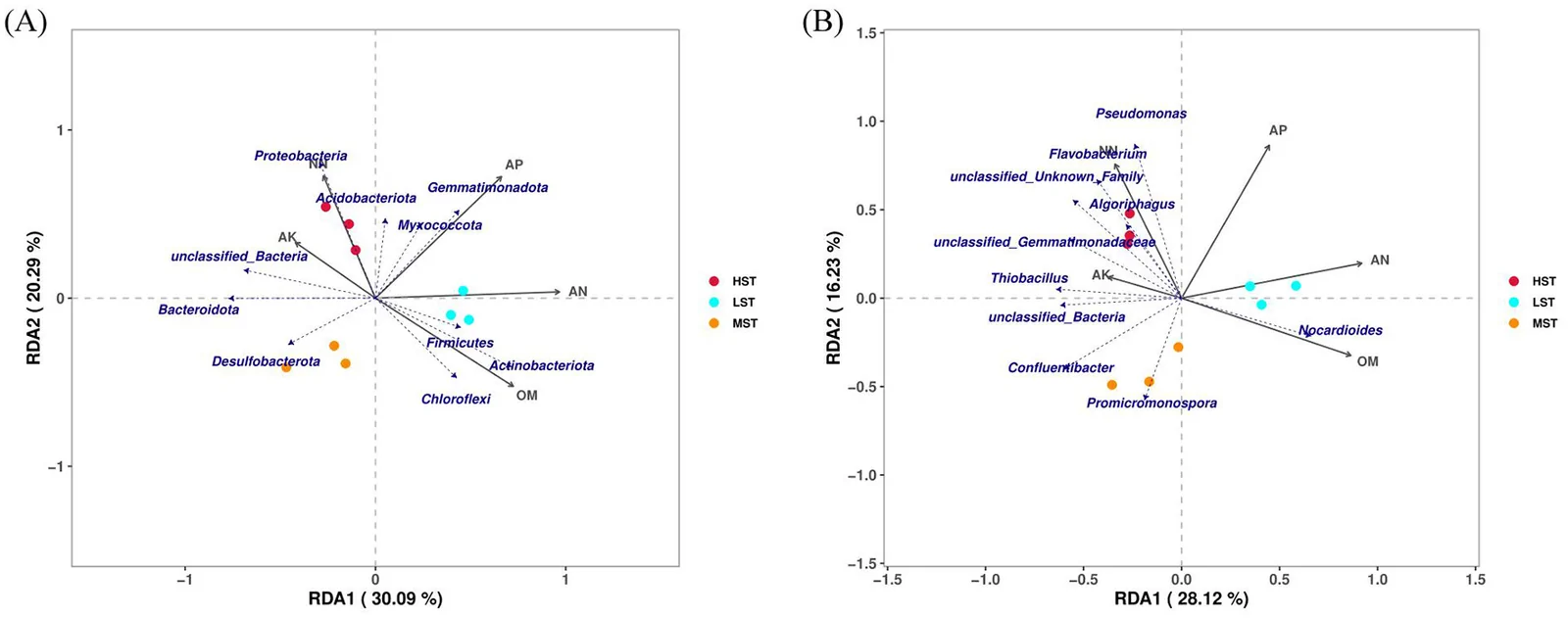
RDA plots showing relationships between environmental factors and bacterial communities **(A)** Phylum level; **(B)** genus level). The horizontal and vertical axes represent the two ordination axes. Colored points denote samples from different groups; points of the same color originate from the same treatment. Gray arrows represent environmental factors; longer arrows indicate stronger influence. The angle between the dashed line connecting a sample/taxon to the origin and the gray arrow indicates correlation: acute angles denote positive correlation, obtuse angles denote negative correlation, and right angles indicate no correlation. Five environmental variables were retained in the RDA model after stepwise VIF screening (all VIF < 10): AP (6.25), AK (1.53), OM (3.29), AN (8.10), and NN (3.67), with no significant multicollinearity. OM, NN, AN, AP, and AK represent organic matter, nitrate-nitrogen, ammonium-nitrogen, available phosphorus and available potassium, respectively.

Redundancy analysis (RDA) revealed clear associations between environmental factors and soil bacterial community structure (Figure 6). At the phylum level, the first principal component (RDA1) explained 30.09% of the total variation, and the second principal component (RDA2) explained 20.29%, with a cumulative explanatory rate of 50.38% (Figure 6A). Among environmental variables, organic matter (OM) significantly affected bacterial community richness (*P* < 0.05), whereas ammonium-nitrogen (AN) and available phosphorus (AP) had extremely significant effects (*P* < 0.01). The bacterial community under low salinity showed the strongest correlations with these factors. AN exerted the greatest influence at the phylum level, explaining 91.51% of the variation in community composition (*R*^2^ = 0.9151, *P* = 0.007). It was positively correlated with the relative abundances of Firmicutes, Actinobacteriota, Gemmatimonadota, Chloroflexi, and Myxococcota. At the genus level, RDA1 and RDA2 explained 28.12% and 16.23% of the total variation, respectively, with a cumulative explanatory rate of 44.35% (Figure 6B). Ammonium-nitrogen (AN), nitrate-nitrogen (NN) and available phosphorus (AP) significantly influenced bacterial community composition (*P* < 0.05), while OM had extremely significant effects (*P* < 0.01). NN was strongly associated with bacterial community structure under high salinity, whereas AP, AN, and OM showed stronger correlations under low salinity. Among these variables, AN had the greatest influence at the genus level, explaining 91.83% of community variation (R^2^ = 0.9183, *P* = 0.007) and showing a positive correlation specifically with the genus *Nocardioides*. Detailed loading values on RDA1/RDA2, explanatory power (R^2^) and significance (*P*-value) of each soil physicochemical factor are listed in Supplementary file 3.

### 3.8 Salinity-responsive bacterial biomarkers via LEfSe

**Figure 7:**
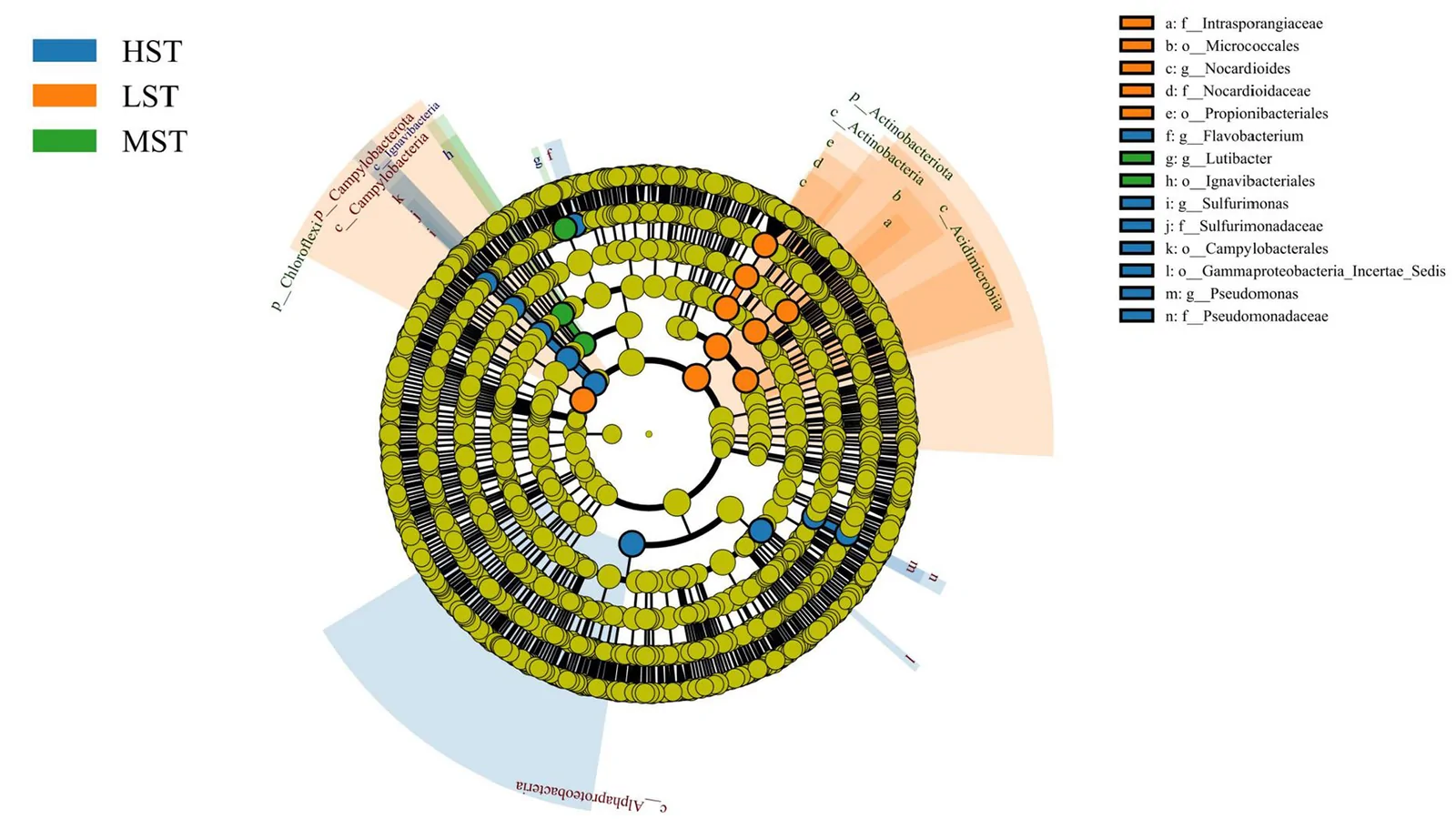
LEfSe analysis showing differentially abundant taxa among treatments. Colored dots represent taxa with significant differences among groups. Concentric circles from inner to outer represent taxonomic levels from phylum to species.

LEfSe analysis (LDA threshold > 4.0, *P* < 0.05) was performed to identify taxa with significant differences among treatments. Distinct microbial biomarkers were identified for each salinity group (Figure 7). Intrasporangiaceae and *Nocardioides* were dominant biomarkers in the low-salinity group; Ignavibacteriales and *Lutibacter* were characteristic of the medium-salinity group; and Alphaproteobacteria, Gammaproteobacteria_Incertae_Sedis*, Flavobacterium, Sulfurimonas*, and *Pseudomonas* were enriched in the high-salinity group. LEfSe biomarker plots covering all taxonomic ranks (from phylum to genus) are provided in Supplementary file 2.

### 3.9 Functional prediction of microbial biomarkers under saline conditions

**Figure 8:**
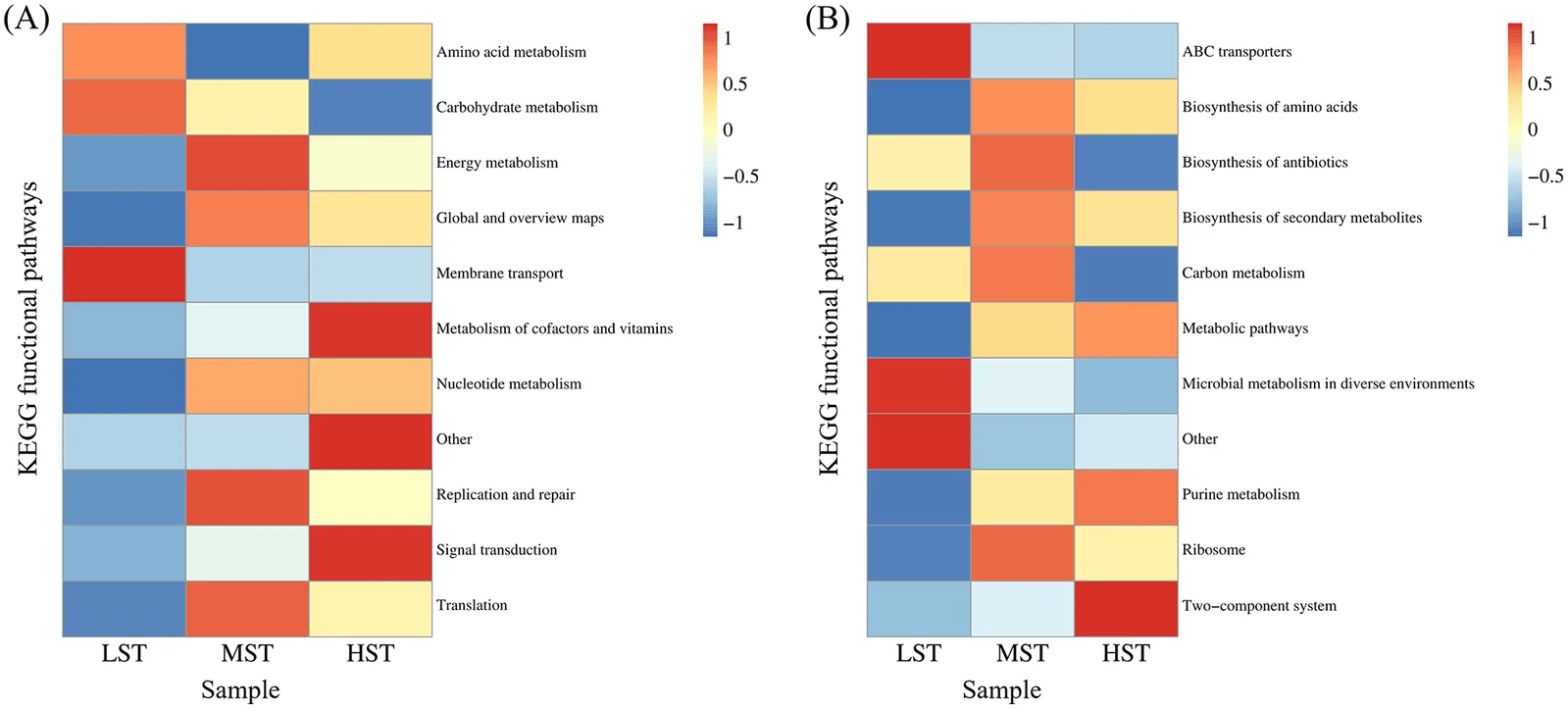
Heatmap of predicted KEGG functional profiles. **(A)** Level 2 pathways; **(B)** Level 3 pathways. Colors represent Z-score-normalized abundance across groups (red, high; blue, low).

Based on the biomarker taxa identified by LEfSe, PICRUSt2 analysis revealed functional differentiation in metabolic potentials across different salinity levels (Figure 8). At the KEGG level 2 pathway level (Figure 8A), functional differentiation was observed among groups. The LST group exhibited higher abundance of Membrane transport and Carbohydrate metabolism pathways. The MST group was characterized by increased abundance in Energy metabolism, Replication and repair, Translation, and Global and overview maps. In contrast, the HST group showed predominant abundance in Metabolism of cofactors and vitamins, Signal transduction, and Other pathways.Further analysis of KEGG level 3 pathways (Figure 8B). The LST group was mainly characterized by higher levels of ABC transporters and Microbial metabolism in diverse environments. Pathways related to Biosynthesis of amino acids, Biosynthesis of antibiotics, Biosynthesis of secondary metabolites, Carbon metabolism, and Ribosome were relatively more abundant in the MST group. The Two-component system, Purine metabolism, and Metabolic pathways exhibited higher relative abundance in the HST group.

### 3.10 Functional prediction of wheat rhizosphere bacterial communities

**Figure 9:**
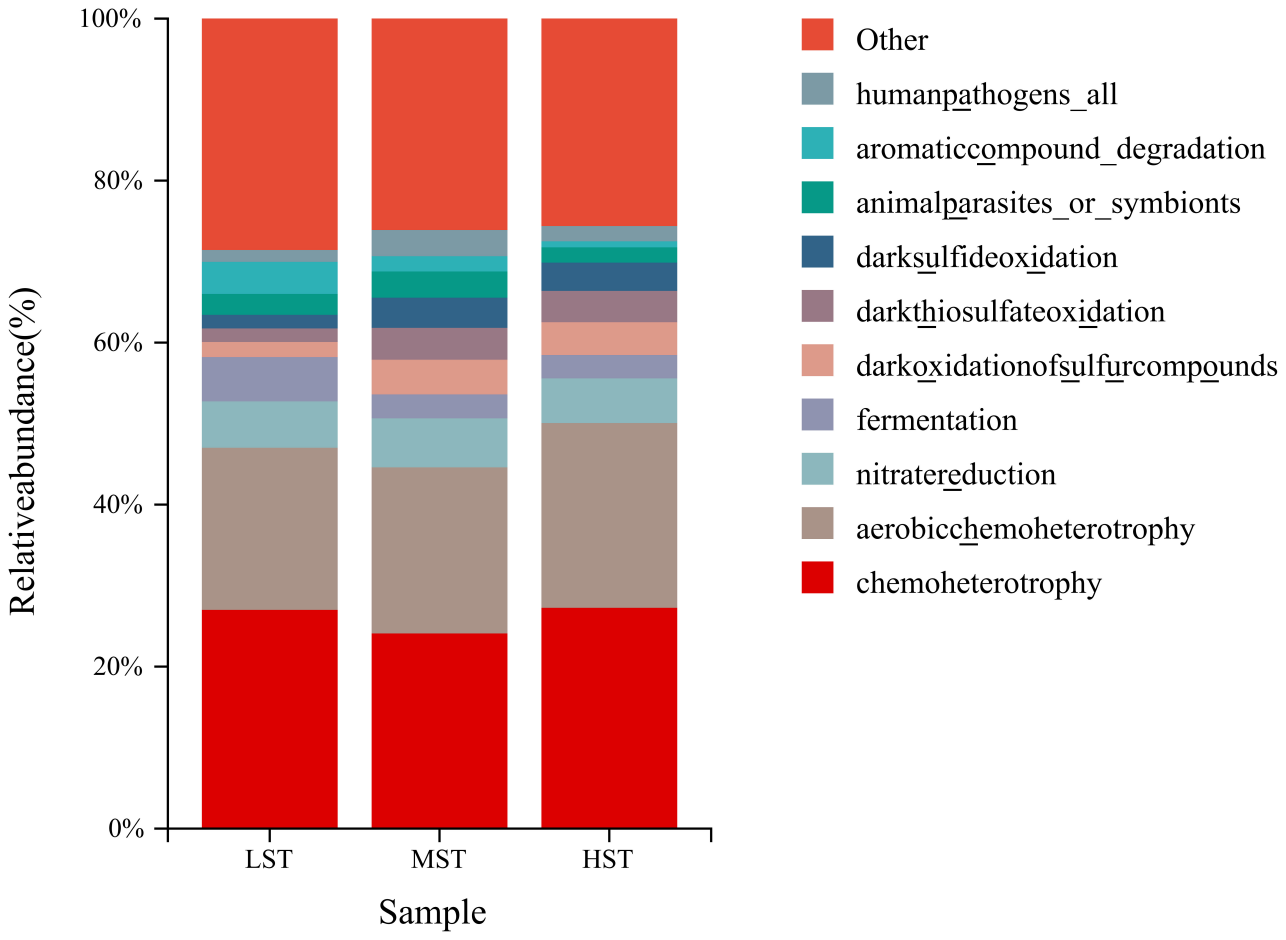
Stacked bar chart showing the relative abundance of the top 10 predicted functional groups of wheat rhizosphere bacterial communities under low-salinity (LST), medium-salinity (MST), and high-salinity (HST) treatments. Functional profiles were annotated using FAPROTAX. The vertical axis represents relative abundance (%). The category “Other” includes all remaining functional groups outside the top 10 entries.

FAPROTAX was used to predict functional profiles of wheat rhizosphere bacterial communities based on established functional databases. Functional categories included chemoheterotrophy, aerobic chemoheterotrophy, nitrate reduction, and others related to nutrient metabolism, environmental adaptation, and host interactions (Figure 9). Under all three salinity treatments, chemoheterotrophy and aerobic chemoheterotrophy were the dominant functional groups. Their combined relative abundance ranged from 47.01% to 50.11%, indicating that these functions may play central roles in supporting material cycling and energy flow within the rhizosphere microbial community. No significant changes were observed in the predicted metabolic pathways of wheat rhizosphere bacterial communities under salinity stress

## 4 Discussion

### 4.1 Coordinated responses to soil physicochemical properties and bacterial communities under salt regulation

Soil salinization poses a serious threat to food security and ecosystem health and has become a major driver of ecosystem degradation worldwide (Tariq et al., 2026). The resulting salt stress inhibits plant growth and development through osmotic imbalance, ion toxicity, and oxidative damage, thereby constraining crop yield improvement (Beillouin et al., 2023). Soil organic matter is the material foundation of soil fertility and an important indicator of fertility status (Hou et al., 2023). In this study, increasing soil salinity significantly reduced organic matter content and thus decreased soil fertility, consistent with the conclusions of Huagang Hou, Chen Meng, and others (Chen et al., 2024; Dong et al., 2023). This may occur because elevated salinity suppresses the growth and metabolic activity of soil microorganisms, thereby directly limiting the synthesis and accumulation of organic matter (Srivastava et al., 2023). The continuous flooding in rice season creates anaerobic conditions that inhibit microbial mineralization and slow organic matter decomposition, partially offsetting salinity-induced losses. In contrast, aerobic wheat-season conditions revive microbial metabolism, intensifying salinity's inhibitory effect and accelerating organic matter depletion (de Freitas et al., 2024; Li et al., 2025). This wetting-drying alternation, combined with salinity stress, drives the significant decline in soil organic matter over the annual rotation. At the same time, salinity stress reduces plant productivity, which can decrease crop residue inputs, root exudation, and the supply of biogenic organic matter to soil.

Distinct from single-crop cultivation, the rice–wheat rotation system features periodic flooding in rice season and aerobic drying during wheat planting, and such alternating hydrological conditions fundamentally modulate rhizosphere nutrient transformation alongside salinity variation (Tao and Liu, 2024). Under rice–wheat cropping in the saline–alkali soils of the Yellow River Delta, salinity significantly regulated nitrogen and phosphorus forms in wheat rhizosphere soil. Ammonium-nitrogen content peaked under low-salinity conditions, whereas nitrate-nitrogen reached its highest value under high-salinity conditions. Salinization influences soil nitrogen cycling through multiple mechanisms rather than a single pathway (Tariq et al., 2026; Tao et al., 2024; Li et al., 2024). We presumed that different bacterial groups involved in nitrogen cycling vary in their sensitivity to salt concentrations, potentially also influenced by fertilization practices. Available phosphorus content was lowest under the medium-salinity treatment, which contrasts with the findings of Li et al. (Li et al., 2024). At moderate salinity, the sharp decline in P is likely primarily attributable to precipitation fixation by Ca^2+^/Mg^2+^; at high salinity (Islam et al., 2023). The lack of further decline is presumably due to Na^+^ displacement and anion competition promoting desorption, while halotolerant microbial lysis and enhanced mineralization might collectively offset precipitation losses (Tariq et al., 2026). In this study, available potassium content remained stable across salinity levels, suggesting that the soil potassium pool at the experimental site has a certain buffering capacity (Kang et al., 2014), which may offset potential potassium leaching and fluctuations in microbial potassium utilization induced by salt stress. Soil enzymes, as biological catalysts, are crucial indicators for evaluating soil quality (Jaiswal et al., 2016). In this study, increasing soil salinity significantly suppressed sucrase activity, consistent with the findings of Rui Li et al. (Li et al., 2024a). This may occur because elevated salinity alters enzyme spatial conformation and reduces the abundance of sucrase-associated phyla such as Actinobacteriota and Gemmatimonas.

In addition, our results indicate that, at the phylum level, ammonium-nitrogen content was closely associated with microbial community structure, showing positive correlation with the relative abundance of Actinobacteriota and Chloroflexi. Previous studies have reported that the distribution of Chloroflexi is significantly correlated with nitrogen indicators such as soil ammonium nitrogen (Li et al., 2024b). Actinobacteriota can secrete diverse extracellular enzymes that promote the mineralization and decomposition of nitrogen-containing organic matter in soil (Tong et al., 2024). At the genus level, ammonium-nitrogen content was closely associated with microbial community structure, showing a positive correlation with the relative abundance of *Nocardia*. *Nocardia* species can secrete multiple hydrolytic enzymes and participate in the decomposition of complex organic matter in soil (Maslov and Maslova, 2021). Ammonium-nitrogen was significantly correlated with soil bacterial community composition, and further targeted fertilization trials are required to verify whether rational ammonium-nitrogen management optimizes rhizosphere bacterial assembly in saline–alkali rice–wheat rotation soils.

It should be noted that redundancy analysis only reveals correlative associations between environmental variables and bacterial communities and cannot quantitatively separate direct and indirect regulatory pathways. Salinity can reshape rhizosphere bacterial communities through two distinct routes. On the one hand, salinity imposes direct stress on microorganisms via ionic toxicity and osmotic pressure, directly filtering and restructuring bacterial assemblages (Rath and Rousk, 2015). On the other hand, salinity acts indirectly by altering soil nitrogen forms, phosphorus availability and soil enzyme activities. These changes modify resource supply and soil microhabitat conditions, which further drive the succession of rhizosphere bacterial communities (Rath and Rousk, 2015; Ren et al., 2026). Rigorous quantitative separation of these direct and indirect effects requires advanced statistical approaches such as structural equation modeling or mediation analysis, which we recommend for future investigations.

### 4.2 Effects of salinity regulation on diversity and structure of wheat rhizosphere bacterial communities

Rhizosphere microorganisms, often referred to as the “second genome” of plants, play pivotal roles in soil substance transformation (Berendsen et al., 2012). This process not only supports plant nutrient acquisition and growth but also substantially enhances stress resistance (Gong et al., 2024). Microbial communities regulate a range of plant stress responses, including those that contribute to salt tolerance (Peng et al., 2023). In this study, wheat rhizosphere bacterial community diversity (Shannon and Simpson indices) progressively decreased with increasing salinity, consistent with the experimental conclusions of Liu et al. (2025). In contrast, community richness (Chao1 and ACE indices) peaked under the medium-salinity treatment, which was consistent with the OTU abundance results and aligned with the Moderate Disturbance Hypothesis (Bao et al., 2020). Under medium-salinity conditions, moderate salt stress may have disrupted the competitive balance among dominant taxa, creating ecological niches for more salt-tolerant microorganisms and thereby increasing community richness. Given that newly colonized salt-tolerant taxa generally maintained low relative abundances, resulting in uneven species distribution within the community (Royle et al., 2026). Such reduced evenness counteracted the positive effect of elevated taxon number, which explains the continuous decline in diversity indices. By contrast, high salinity likely exceeded the tolerance thresholds of many microorganisms, leading to the loss of sensitive groups and the persistence of only a limited number of salt-tolerant taxa (Rahman et al., 2025). The combined loss of both rare and moderately abundant taxa resulted in marked declines in both richness and diversity. Moreover, analysis of shared OTUs among treatments showed that the medium- and high-salinity groups shared substantially more OTUs than the medium-low or low-high combinations, consistent with the findings of Wang et al. (2021). This suggests that the medium-salinity environment represents a transitional state along the salinity gradient (Zhi et al., 2026; Wang et al., 2018). Its community structure may retain taxa adapted to low-salinity conditions while selecting for those capable of persisting under high salinity, thereby exhibiting connectivity with both low- and high-salinity environments. Early comprehensive studies have identified salinity as a primary environmental factor shaping microbial diversity across global ecosystems (Tariq et al., 2026). Beta diversity analysis in the present study supports this conclusion, indicating that differences in salinity treatment concentration acted as the principal driver of structural variation in the wheat rhizosphere microbial community.

In this study, Proteobacteria, Bacteroidota, Actinobacteriota, Chloroflexi, and Acidobacteriota were the dominant bacterial phyla across all treatments, which are well-recognized as typical taxa in the wheat rhizosphere (Fouad et al., 2025). This observation is consistent with the conclusions reported by Jiao Zhao et al. regarding the rhizosphere microenvironment of saline–alkali soils in the Yellow River Delta (Zhao et al., 2020). With increasing salinity, the relative abundances of Proteobacteria and Bacteroidota increased, whereas Actinobacteriota decreased. This pattern partially aligns with the results reported by Fouad et al. (2025) and may be attributable to differential sensitivities of taxa within Actinobacteriota to saline conditions. Proteobacteria exhibited the highest relative abundance across all treatments, and increasing salinity markedly enhanced its abundance. Previous studies have shown that this phylum includes numerous nitrogen-fixing groups and exhibits strong environmental adaptability, resulting in relatively stable distributions and abundances under changing conditions (Liu et al., 2014). Moreover, Proteobacteria encompasses many halophilic bacterial taxa and includes dominant groups in both primary and secondary saline–alkali soils (Canfora et al., 2014). For Bacteroidetes, relative abundance increased with salinity. Studies have demonstrated that salt stress promotes the enrichment of this phylum in wheat rhizosphere soils, and its nitrogen-fixing capacity can further support plant growth through nutrient provision (Peng et al., 2023; Zhang et al., 2025). In addition, Bacteroidetes exhibits strong tolerance to high-salinity environments and is a typical dominant phylum in saline–alkali soils (Niu et al., 2017). From the perspective of rice–wheat rotation legacy, the enrichment of Proteobacteria and Bacteroidetes under high salinity also reflects their adaptability to fluctuating redox conditions: both phyla contain abundant facultative anaerobic taxa that thrive under alternating flooded–dry habitats, which differentiates the community composition of this rotation system from upland saline soils dominated by strictly aerobic halophilic taxa. Acidobacteriota showed stable relative abundance across all treatments in this study. Previous research indicates that Acidobacteriota constitutes a relatively stable microbial group characterized by low nutrient requirements and consistent resource utilization (Duineveld et al., 1998). Its oligotrophic lifestyle may enable gradual adaptation to changes in the rhizosphere environment, including salinity-induced shifts in soil microhabitats (Du et al., 2016). The phyla represented by the top ten abundant genera were dominant in the wheat rhizosphere, and their trends with increasing salinity were consistent with those observed at the phylum level.

Notably, at the genus level, unclassified Bacteria showed the highest relative abundance across all treatments and increased with salinity, suggesting a potential role in wheat salt stress responses that warrants further investigation. These unclassified bacteria likely adopt the classic “salt-out” or “salt-in” strategies to maintain intracellular osmotic homeostasis. According to metagenomic investigations of microbial communities along natural salinity gradients, halotolerant bacteria in most environments tend to cope with hyperosmotic conditions by synthesizing and accumulating compatible solutes (Gao et al., 2024; Wu et al., 2024). As a relatively underexploited natural habitat, the saline-alkali region of the Yellow River Delta harbors a wealth of unclassified and novel microbial resources that are not fully represented in current taxonomic databases (Li et al., 2026). The high relative abundance of unclassified bacteria also reflects inherent limitations of 16S rRNA amplicon-based taxonomic annotation (Bars-Cortina et al., 2023). Further research should integrate bacterial isolation and genomic analysis: salinity-gradient culturing can characterize salt-associated taxa, while metagenomics or single-cell genomics refine taxonomy and reveal metabolic potential and salt-adaptation mechanisms.

Previous studies have shown that certain halotolerant bacteria within Gammaproteobacteria and Alphaproteobacteria can help non-host plants tolerate saline–alkali conditions (Yuan et al., 2016). Our LEfSe results indicate that Gamma-Proteobacteria and Alpha-Proteobacteria played key roles in the high-salinity group. Among the taxa enriched under high salinity, *Pseudomonas* is recognized as a halotolerant microorganism with plant growth-promoting properties (Yadav and Saxena, 2018); *Flavobacterium* is a beneficial microbe that can promote plant growth, suppress plant diseases, and enhance tolerance to abiotic stress (Seo et al., 2024); and *Sulfurimonas* functions as an important driver of rhizosphere sulfur cycling, linking soil insoluble sulfur pools with the nutritional demands of wheat (Gahan and Schmalenberger, 2014). It is therefore presumed that these genera may contribute to wheat resistance under high-salinity stress, which merits further investigation. In addition, *Ignavibacteriales* is widely reported to occur in paddy soils (Bei et al., 2021), and our LEfSe analysis identified it as a biomarker in the medium-salinity group. This may indicate that the rice–wheat cropping context contributed to the selection of key bacterial communities under salinity stress.

### 4.3 Comprehensive analysis of metabolic mechanisms of microbial biomarkers and rhizosphere community functional characteristics

Combined with the KEGG functional enrichment results of microbial biomarkers, we further interpreted the adaptive metabolic strategies of rhizosphere microorganisms under salinity gradients. The two-component system pathway was significantly upregulated under high-salinity stress. As the core pathway for microorganisms to sense extracellular osmotic pressure and regulate ion homeostasis, its high enrichment serves as a vital adaptive mechanism for microbial communities to cope with hyperosmotic stress (Kenney and Anand, 2020). Meanwhile, high salinity markedly suppressed pathways related to energy metabolism, amino acid biosynthesis and carbon skeleton synthesis. This phenomenon indicates that microorganisms actively reduce energy-consuming growth and metabolic processes to resist salt stress and preferentially allocate energy to stress-resistant functions such as osmotic regulation and signal perception (Wang et al., 2024; Kumar et al., 2020).

Based on FAPROTAX functional prediction analysis, the functional composition of rhizosphere bacteria remained relatively stable across salinity treatments, suggesting that although bacterial taxonomic composition differed among groups, communities with similar predicted functions were present in wheat rhizosphere soils. This is consistent with the findings of Chao Yang et al. (Yang et al., 2021). This pattern may be attributable to the high functional redundancy typically observed in soil microbial communities (Grzadziel, 2017). Specifically, as salinity increases, salt-sensitive taxa may decline, whereas salt-tolerant or halophilic taxa may increase in abundance or activity, thereby replacing the former while maintaining similar functional roles.

### 4.4 Limitations of 16S-based functional prediction and outlook for subsequent verification

All functional annotations in this study rely on predictive analysis of 16S rRNA gene sequences, rather than direct metatranscriptomic or metabolomic measurements, which may introduce prediction bias (Bars-Cortina et al., 2023). Furthermore, the potential salt-tolerant functions of key biomarker genera and unclassified bacterial populations inferred from amplicon correlation analysis cannot be fully validated using the current sequencing data. Further studies including *in vitro* pure culture, pot inoculation experiments and multi-omics analysis are needed to verify the actual physiological functions of these key microbes under field saline–alkali conditions. In follow-up work, we will also incorporate ITS-based fungal community analysis to systematically dissect the response patterns of both bacterial and fungal communities to salinity regulation, and achieve a holistic understanding of the saline soil microbial ecosystem under rice-wheat rotation.

## 5 Conclusions

This study collected wheat rhizosphere soils under different salinity treatments in the rice-wheat cropping area of the Yellow River Delta to analyze bacterial community composition, community characteristics, predicted functional profiles, and relationships between microbial communities and soil physicochemical properties. Salinity acted as a primary driver of community assembly, mediating bacterial richness and diversity and triggering gradual community succession rather than abrupt compositional turnover. Ammonium nitrogen was significantly correlated with soil bacterial community composition. Multiple salt-enriched genera, including *Flavobacterium, Sulfurimonas* and *Pseudomonas*, as well as unclassified bacterial taxa, constituted promising microbial resources linked to salt adaptation. Despite distinct taxonomic shifts across salinity levels, the rhizosphere microbiome maintained functional stability, reflecting strong functional redundancy under salt stress. The findings of this study can provide theoretical references for field management of saline farmlands under rice-wheat cropping system. This work clarifies the gradient response characteristics of rhizosphere microorganisms to salinity in this rotation system, suggesting that differentiated management corresponding to varying soil salinity levels combined with nitrogen regulation to optimize the rhizosphere microenvironment is more suitable for wheat production under rice-wheat rotation. The salinity-enriched bacterial genera identified in this study can serve as candidate resources for exploring salt-tolerant microbial strains and developing microbial amendments for wheat in saline soils. Future research should further characterize the biological functions of unclassified bacteria and highly saline-enriched functional microbes and clarify the mechanisms by which key microbial communities mediate salt tolerance in wheat. Field trials should be conducted to verify the efficacy of microbial regulation, nutrient optimization and rotation improvement practices, so as to refine the microecological improvement technical system for saline farmlands under rice-wheat rotation and promote the sustainable utilization of saline land under rice-wheat rotation systems in the Yellow River Delta.

## Data Availability

The datasets presented in this study can be found in online repositories. Raw sequence data were deposited in the NCBI SRA database under BioProject PRJNA1439770 and Biosample numbers SAMN56592217-SAMN56592225.

## Supplementary material

**Supplementary file 1**

Relative abundance of the top 10 bacterial taxa at the phylum and genus levels in wheat rhizosphere soil under low-salinity (LST), medium-salinity (MST), and high-salinity (HST) treatments.

**Supplementary file 2**

A collection of relative abundance bar plots for key bacterial biomarker taxa identified by LEfSe analysis, covering multiple taxonomic levels (phylum, class, order, family, and genus) in wheat rhizosphere soil under low-salinity (LST), medium-salinity (MST), and high-salinity (HST) treatments.

**Supplementary file 3**

Statistical results of redundancy analysis (RDA) showing the loading values (RDA1, RDA2), explanatory power (R^2^), and significance (*P*-value) of soil physicochemical factors (pH, AP, AK, OM, AN, NN) in shaping the rhizosphere bacterial community structure under different salinity treatments.
