# Supplementary material for: Salinity gradients shape rhizosphere bacterial diversity and assembly of wheat in saline-alkali rice–wheat rotation soils
Source: Front Microbiol. 2026 Aug 28;17:1931712. doi: 10.3389/fmicb.2026.1931712 (PMC13561905; doi:10.3389/fmicb.2026.1931712)
Supplement: Supplementary file 2 [file Data_Sheet_2.zip › Supplementary information 2/1_f__Sulfurimonadaceae.pdf]

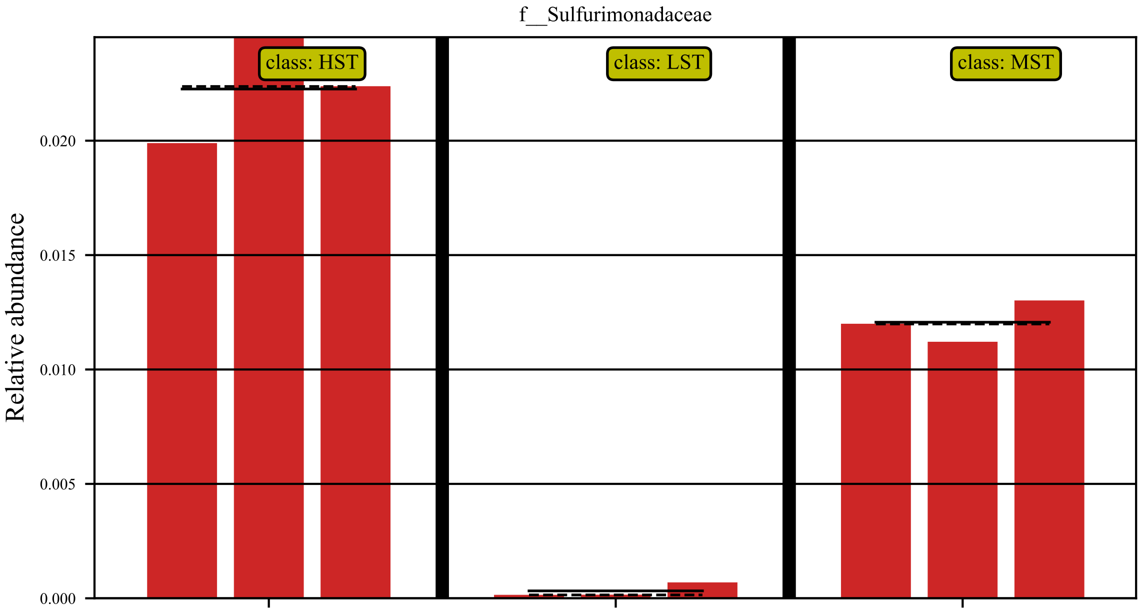

f__Sulfurimonadaceae
class: HST
class: LST
class: MST
Relative abundance
0.020
0.015
0.010
0.005
0.000
