# Supplementary material for: Salinity gradients shape rhizosphere bacterial diversity and assembly of wheat in saline-alkali rice–wheat rotation soils
Source: Front Microbiol. 2026 Aug 28;17:1931712. doi: 10.3389/fmicb.2026.1931712 (PMC13561905; doi:10.3389/fmicb.2026.1931712)
Supplement: Supplementary file 2 [file Data_Sheet_2.zip › Supplementary information 2/1_p__Chloroflexi.pdf]

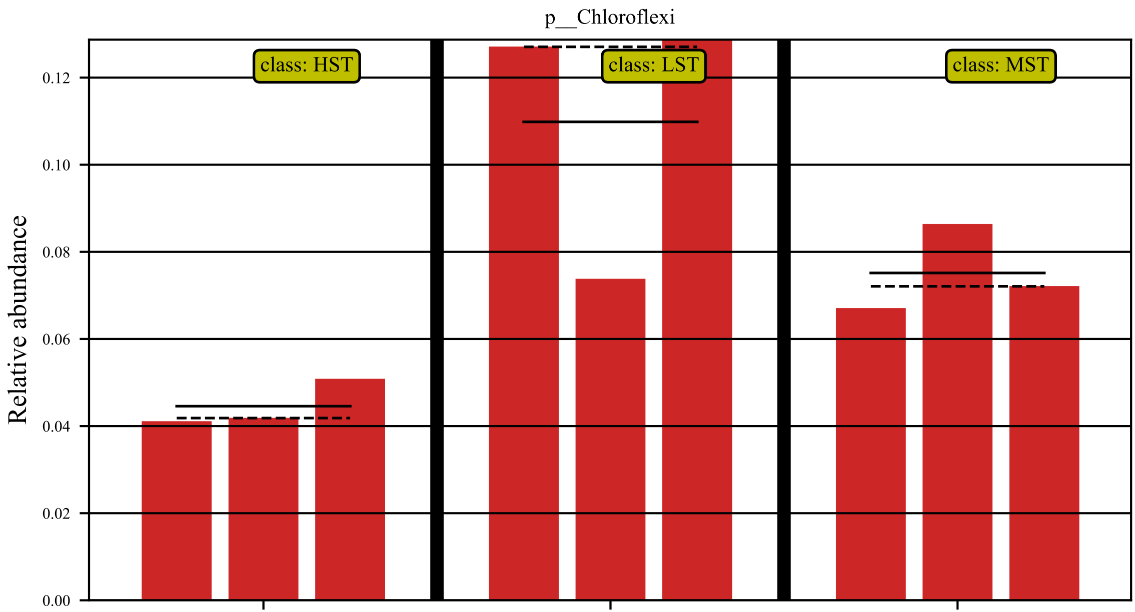
p__Chloroflexi
Relative abundance
0.00
0.02
0.04
0.06
0.08
0.10
0.12
class: HST
class: LST
class: MST
